# Staged Limb Reconstruction Using Ilizarov Fixator in an Infected Tibia Nonunion: A Case Report

**DOI:** 10.7759/cureus.67112

**Published:** 2024-08-18

**Authors:** Shrikrishna A Rakhunde, Sushil Mankar, Nilesh Joshi, Pallav P Agrawal, Vismay V Harkare

**Affiliations:** 1 Orthopedics, N. K. P. Salve Institute of Medical Sciences and Research Centre and Lata Mangeshkar Hospital, Nagpur, IND; 2 Orthopedics and Traumatology, N. K. P. Salve Institute of Medical Sciences and Research Centre and Lata Mangeshkar Hospital, Nagpur, IND

**Keywords:** high-energy trauma, external fixator, refracture, ilizarov fixator, interlocking nail, nonunion

## Abstract

Road traffic accidents are responsible for most lower limb compound fractures. Such fractures have to be treated immediately with utmost care and precision. Patients are sometimes inadequately treated with traditional practices which causes further disability to the patient and makes it more difficult for the orthopedic surgeon. This case report highlights the meticulous planning and management of a distal tibia-fibula-infected nonunion which was initially mal-treated by an unqualified practitioner following trauma on multiple occasions.

## Introduction

Lower limb injuries following high-energy trauma like road traffic accidents, fall from height, gunshot injuries, assaults, etc. often present to the orthopedic surgeon with complex challenges like contamination, bone loss, severe soft tissue insult, and neurological and vascular trauma [1]. These cases often pose hurdles in management by the orthopedic surgeon [2]. For good outcomes, such cases have to be managed as soon as possible with utmost care [3]. Compound fractures with contamination, soft tissue injury, and bone loss have to be treated immediately with thorough debridement and adequate immobilization using external fixators to reduce the chances of infection [4].

In cases where early treatment is not administered, the rate of complications like infection, nonunion, bone gaps, and soft tissue contractures develops, further compromising the patient's condition [5]. The treatment duration is usually prolonged in neglected cases and involves multiple staged surgeries for adequate results [6]. Such patients require a multicentric treatment strategy which includes the orthopedic surgeon, plastic surgeon, and physiotherapist [7].

This case report outlines the management of a 45-year-old male with a neglected tibia nonunion managed with multiple staged surgeries and underscores the complexities involved in managing an infected nonunion tibia with a huge bone gap. We also emphasize the critical role of an interdisciplinary approach in optimizing patient care.

## Case presentation

**Figure 1:**
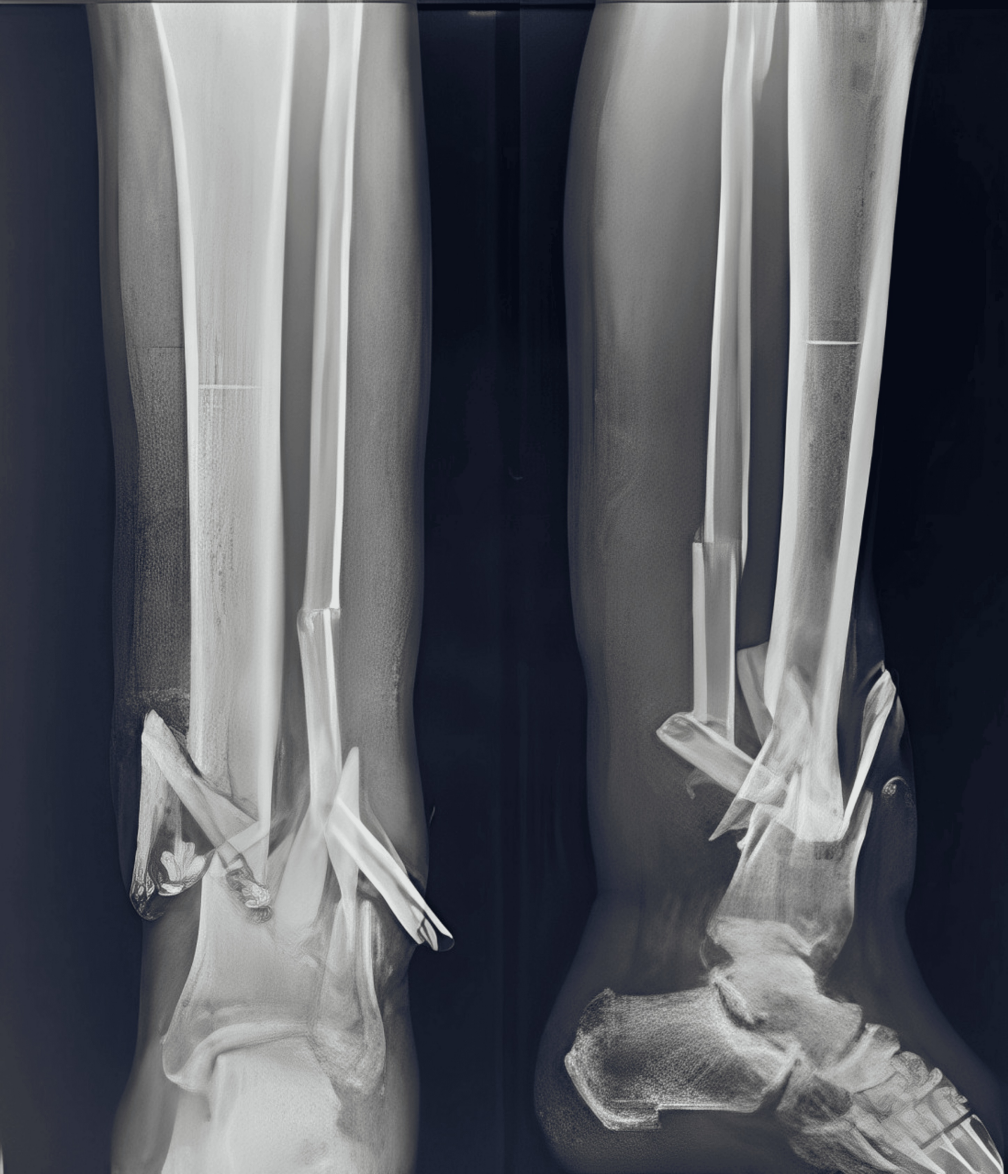
X-ray showing the distal third tibia-fibula fracture with severe communition

**Figure 2:**
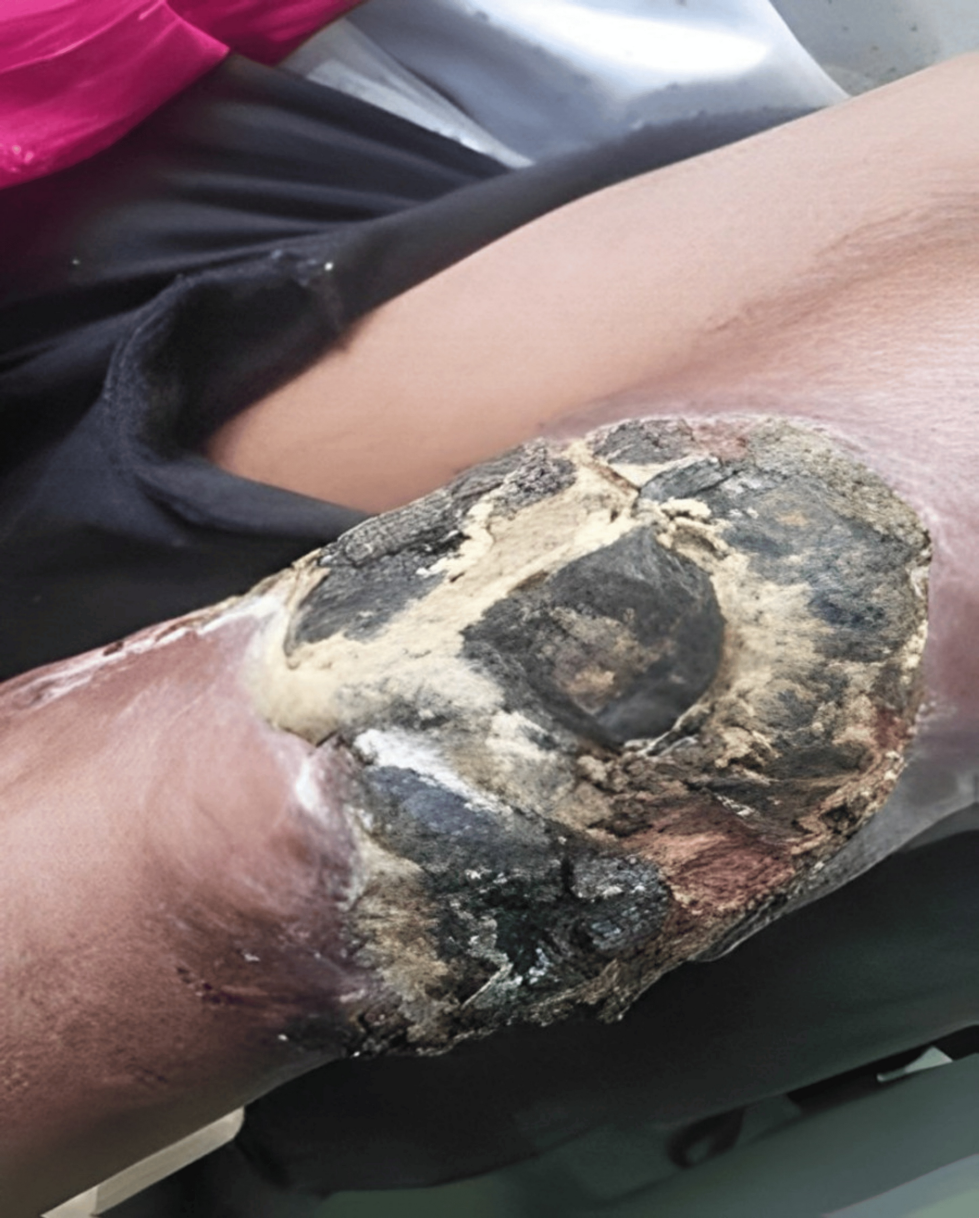
Clinical image of the left leg showing the condition of the affected leg with discharge from the fracture site

A 45-year-old male presented to the outpatient department with a comminuted left tibia-fibula fracture, accompanied by severe soft tissue compromise at the fracture site (see Figure 1 and Figure 2). The patient experienced painful movement and purulent discharge from the fracture site.

The patient was involved in a road traffic accident seven months prior, following which he received an initial diagnosis and care at a primary health center. He was diagnosed with a tibia-fibula fracture and referred to a higher center for surgical management. However, the patient declined surgical intervention and opted for immobilization with an above-knee slab. Subsequently, he did not attend further follow-up appointments, neglecting his condition for three months.

Unfortunately, after this period, the patient experienced another trauma from falling off a wall while asleep. This incident impacted his already fractured leg, exacerbating the injury and compromising the soft tissue, resulting in a compound fracture. The patient sought treatment from an unqualified practitioner (commonly known as a quack), who administered unconventional local treatments including homemade applications and improper splinting with wooden sticks. This treatment regimen continued for five months without supervision from a trained surgeon.

**Figure 3:**
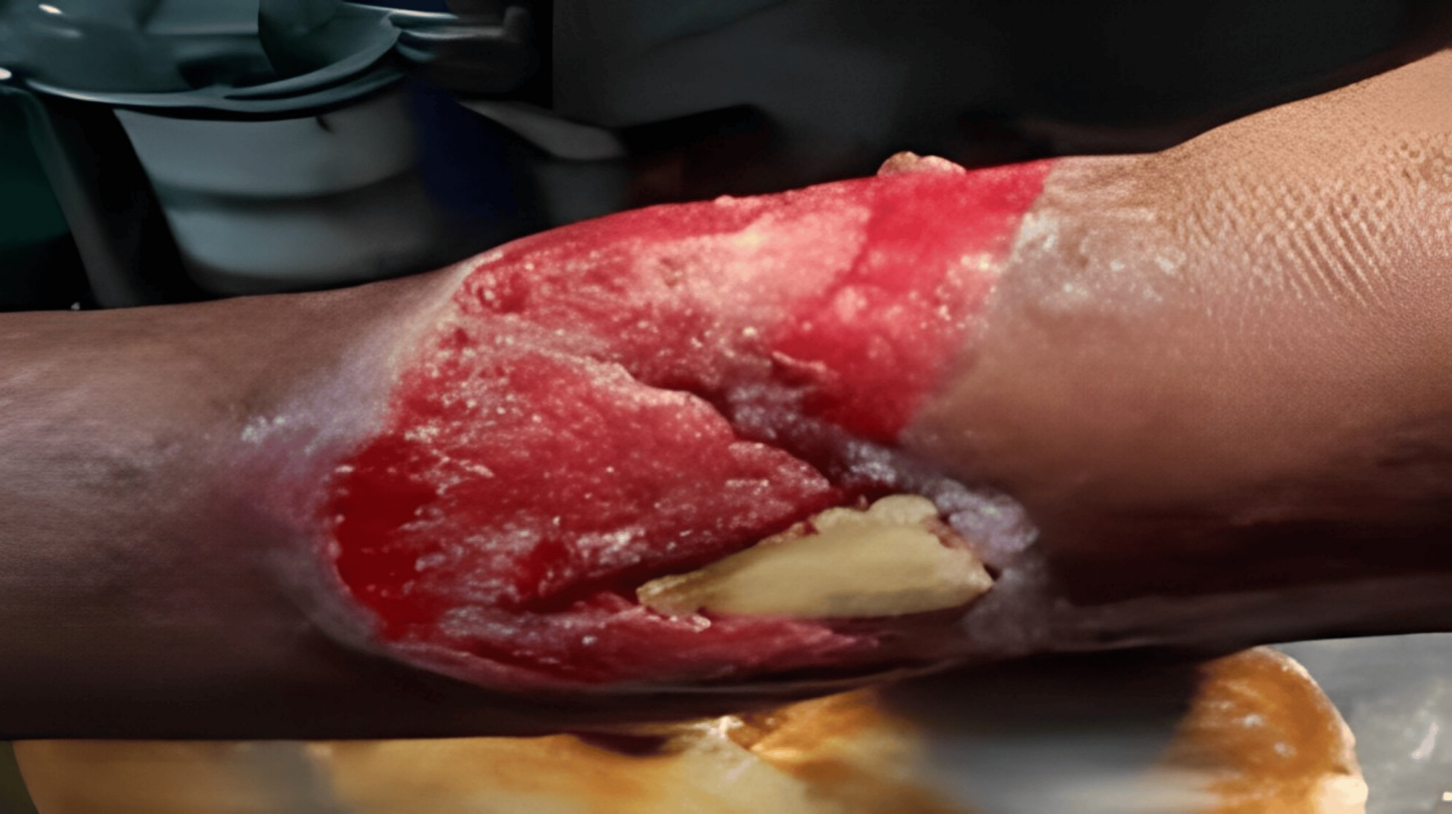
Clinical image post-debridement showing the exposed devitalized bone and significant soft tissue injury

After a total duration of seven months from the initial accident, the patient presented to the outpatient department with the aforementioned issues. He was admitted for further evaluation and preoperative planning. Necessary hematological and radiological investigations were conducted to facilitate surgical planning. The patient underwent thorough debridement and application of an external fixator for the stabilization of the nonunion site (see Figure 3).

**Figure 4:**
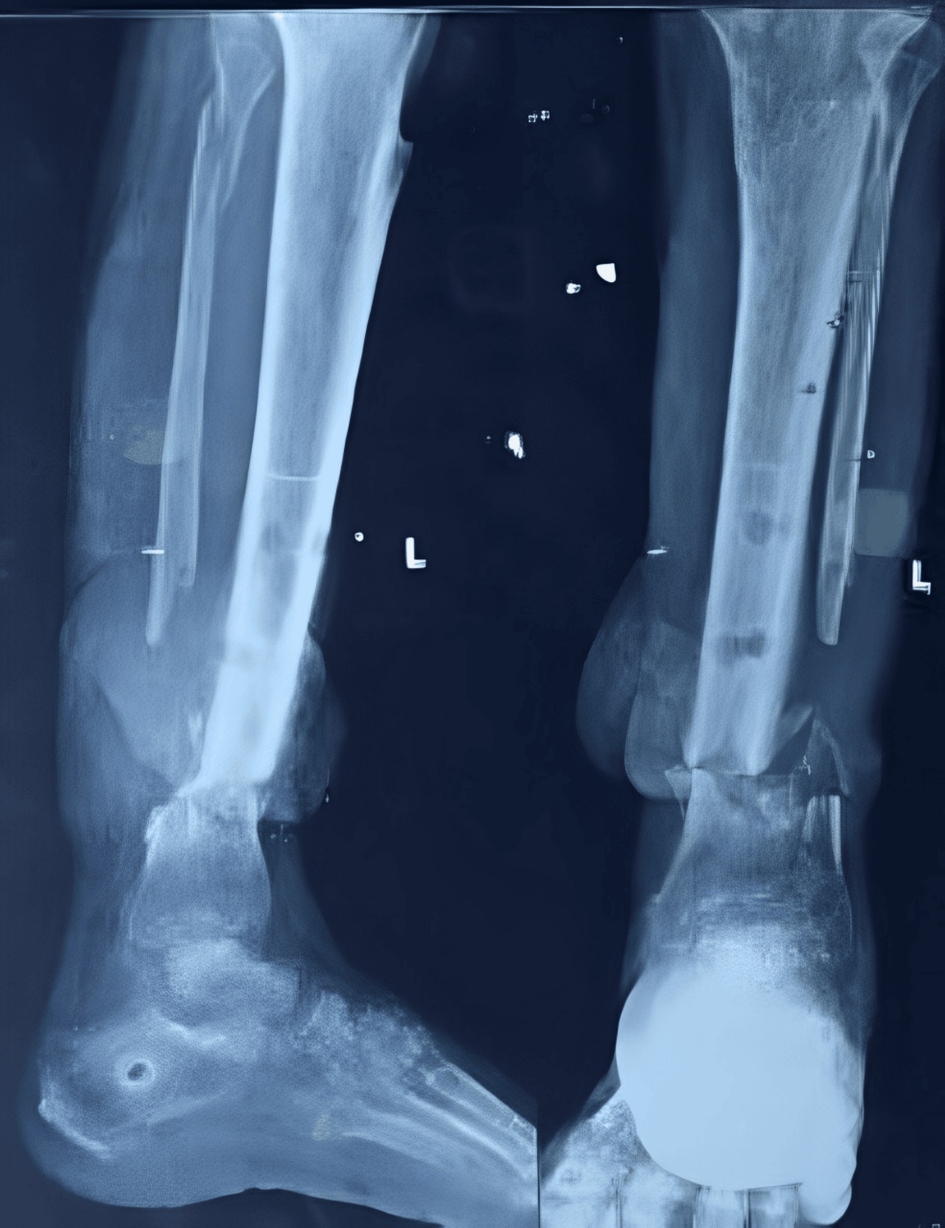
X-ray showing the nonunion at the distal tibia-fibula after the removal of the external fixator

A plastic surgery consultation was conducted, and the patient underwent a deep reverse sural flap procedure to manage the soft tissue damage. Six weeks later, after the flap had healed, the patient was scheduled for definitive surgical intervention. The temporary external fixator was removed (Figure 4).

**Figure 5:**
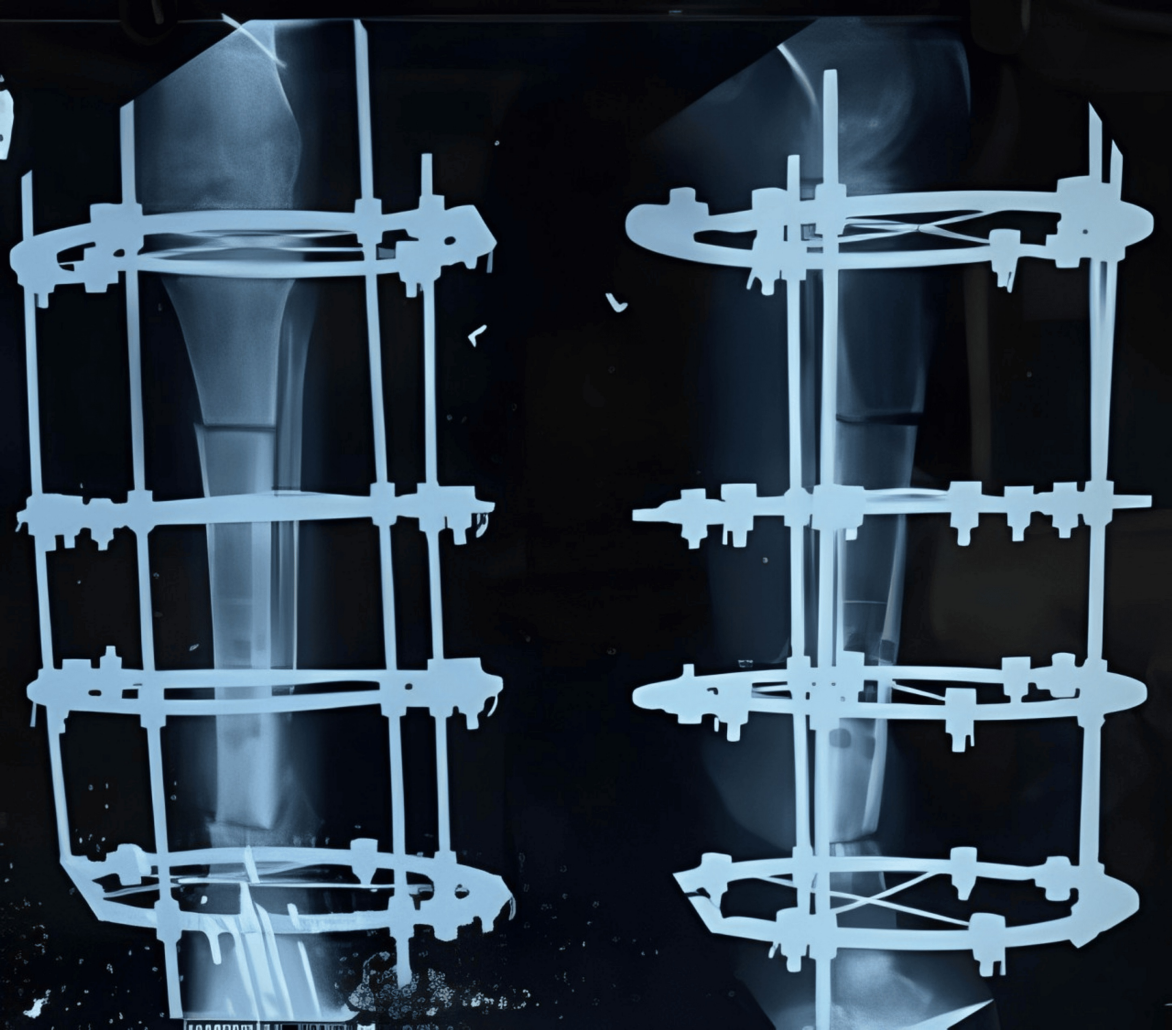
X-ray showing the Ilizarov ring fixator with corticotomy at the proximal site and docking at the distal nonunion site

There was a significant bone loss, resulting in a 7 cm shortening. No obvious clinical signs of infection were present. Additionally, repeat hematological investigations were conducted to confirm the absence of infection before planning the definitive Ilizarov surgery. The Ilizarov fixator was applied for docking at the distal tibia-fibula site, and a corticotomy was performed at the proximal tibia to address the shortening (Figure 5).

**Figure 6:**
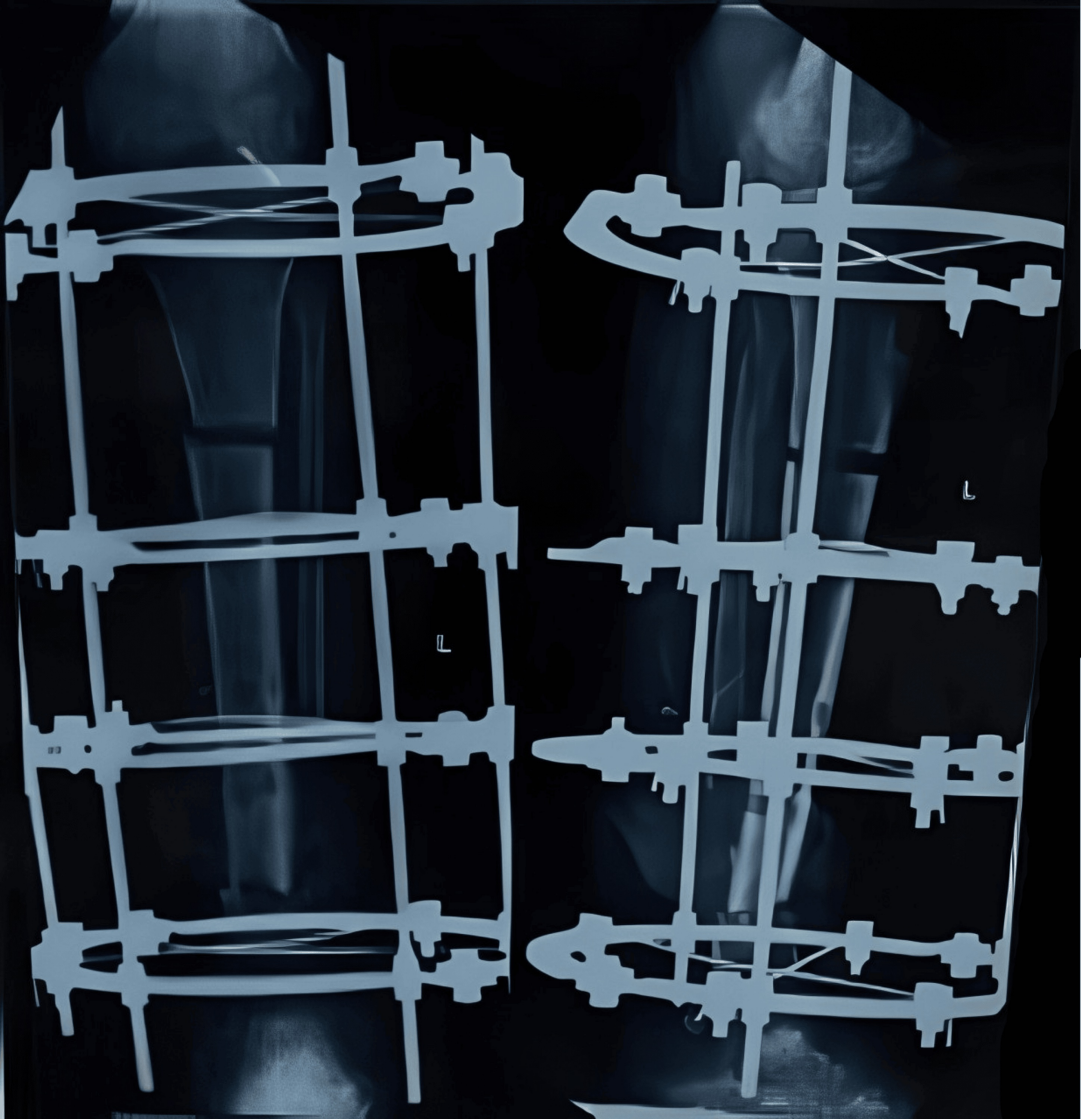
X-ray after one week of distraction at the proximal tibia

**Figure 7:**
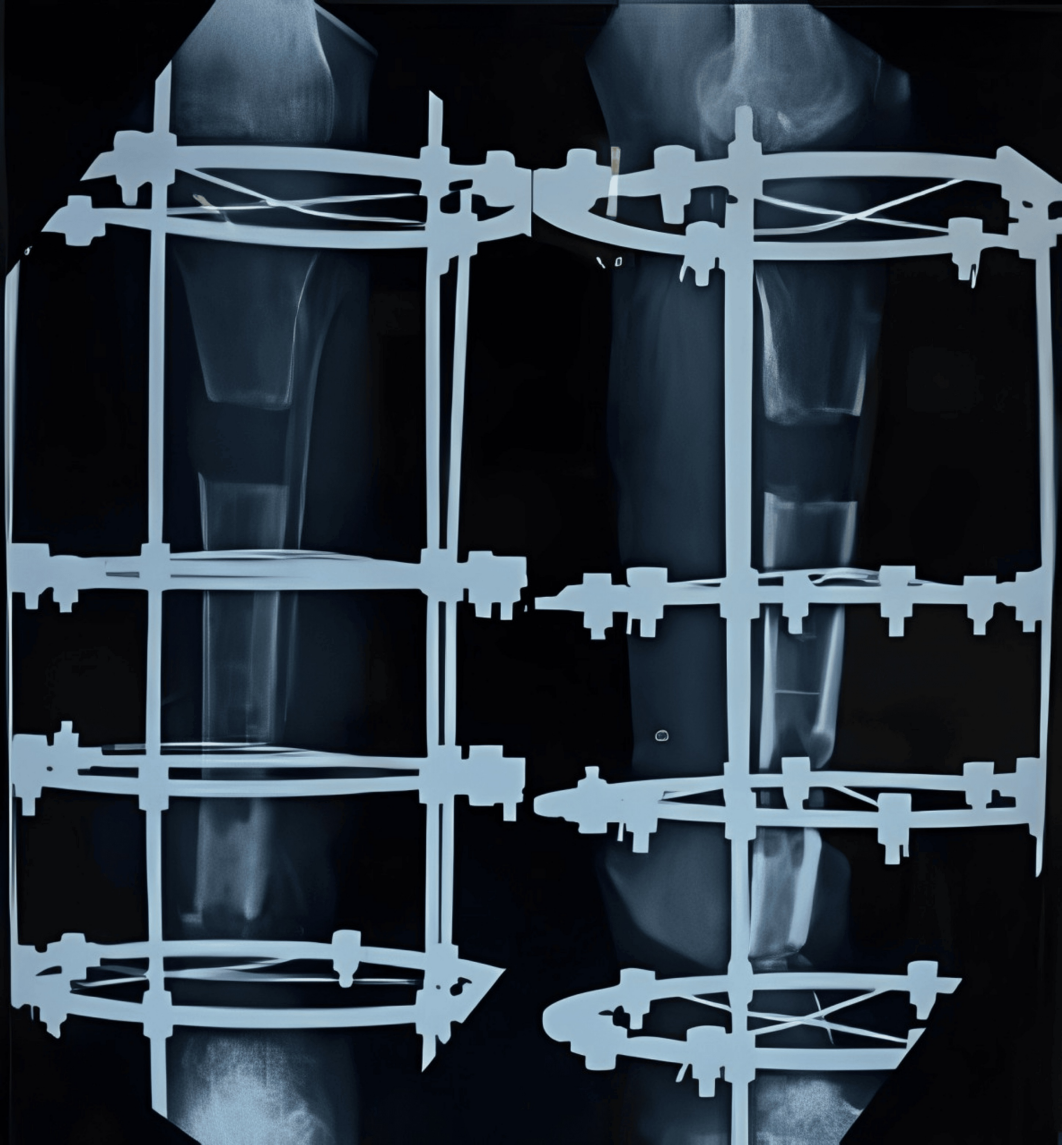
X-ray after eight weeks of distraction

**Figure 8:**
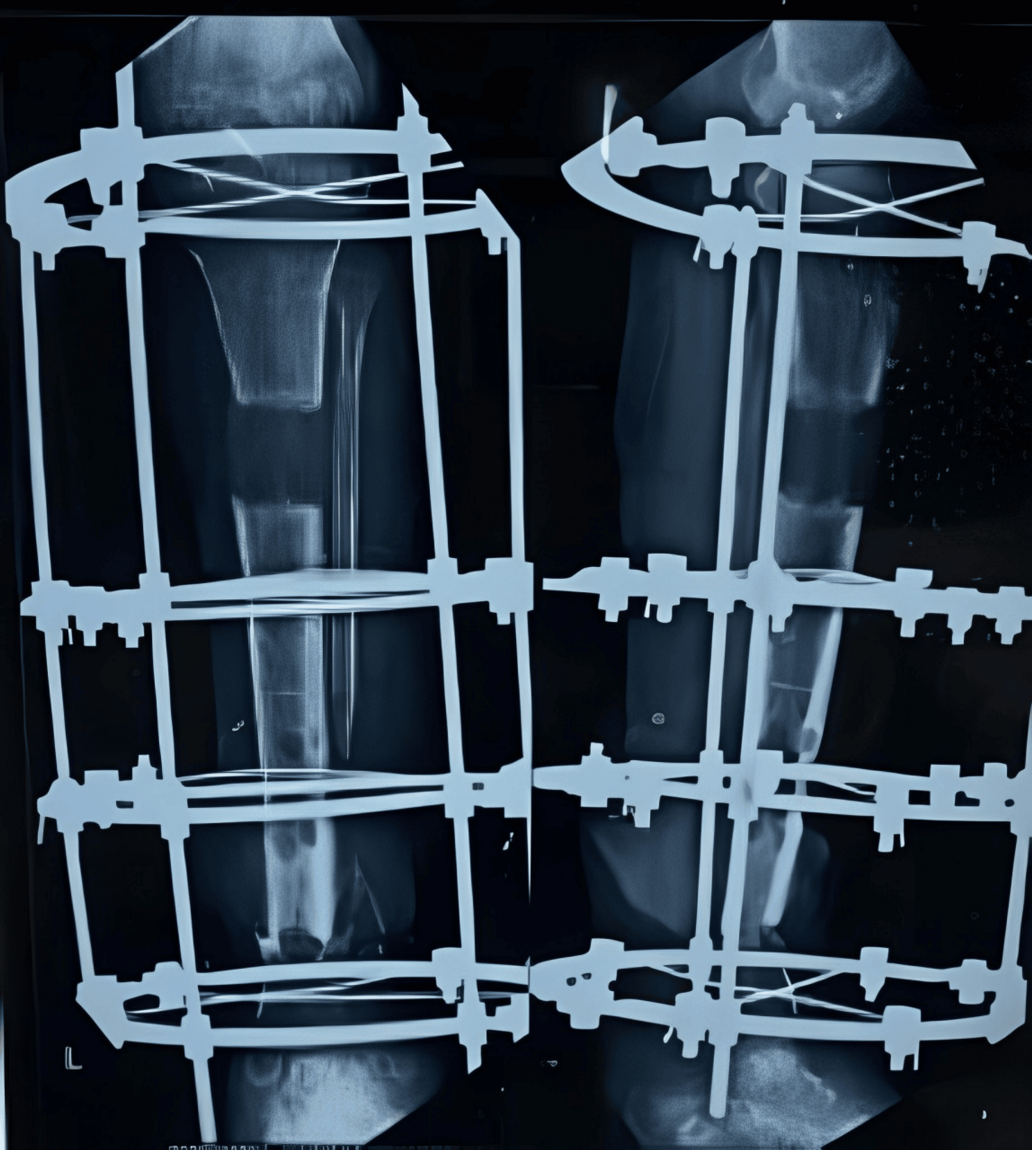
X-ray after three months of distraction

Sequential X-rays were done to observe the correct lengthening of the tibia and to check for union at the distal site (Figures 6-8).

**Figure 9:**
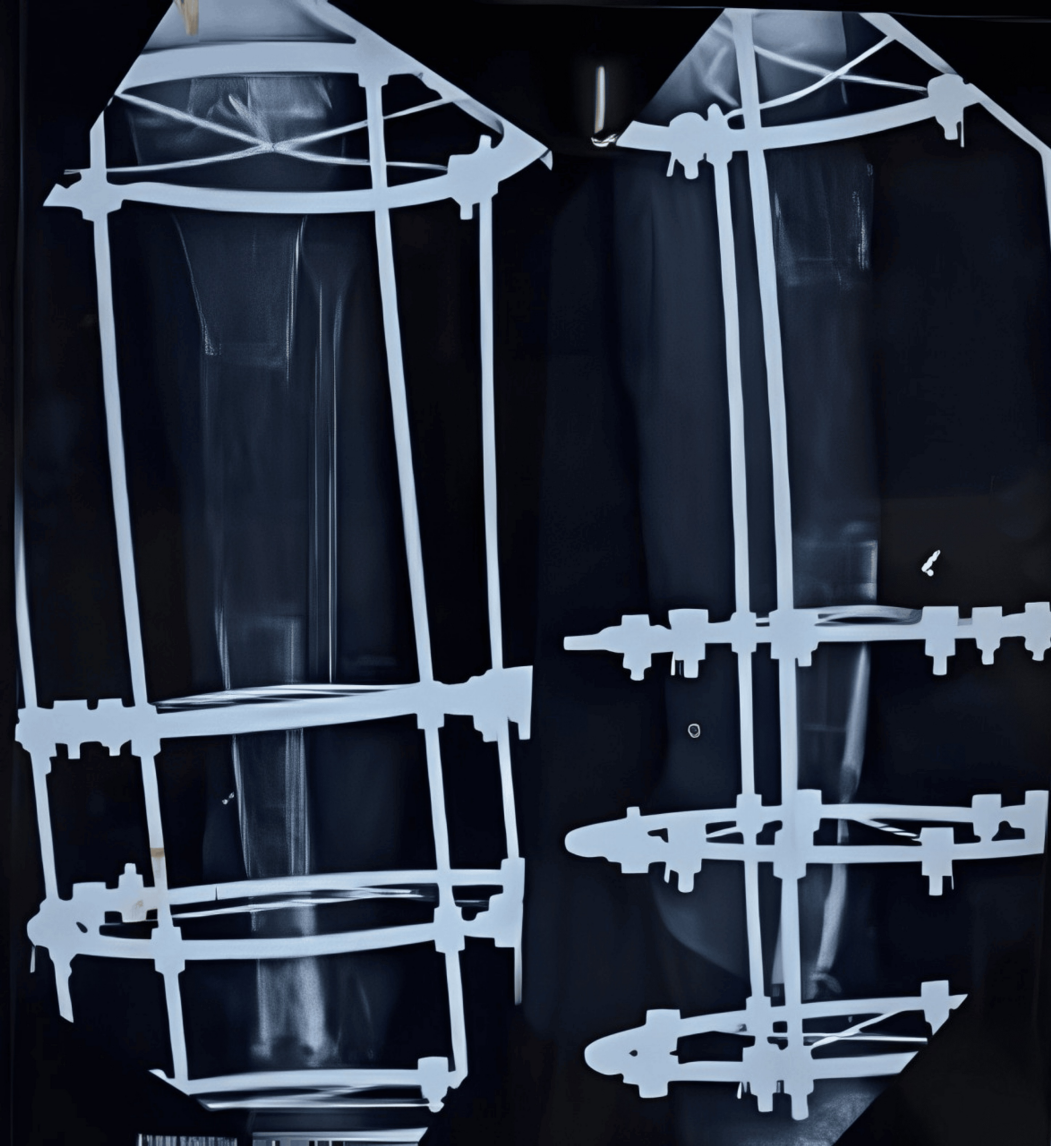
X-ray showing the completed distraction (at the end of six months)

**Figure 10:**
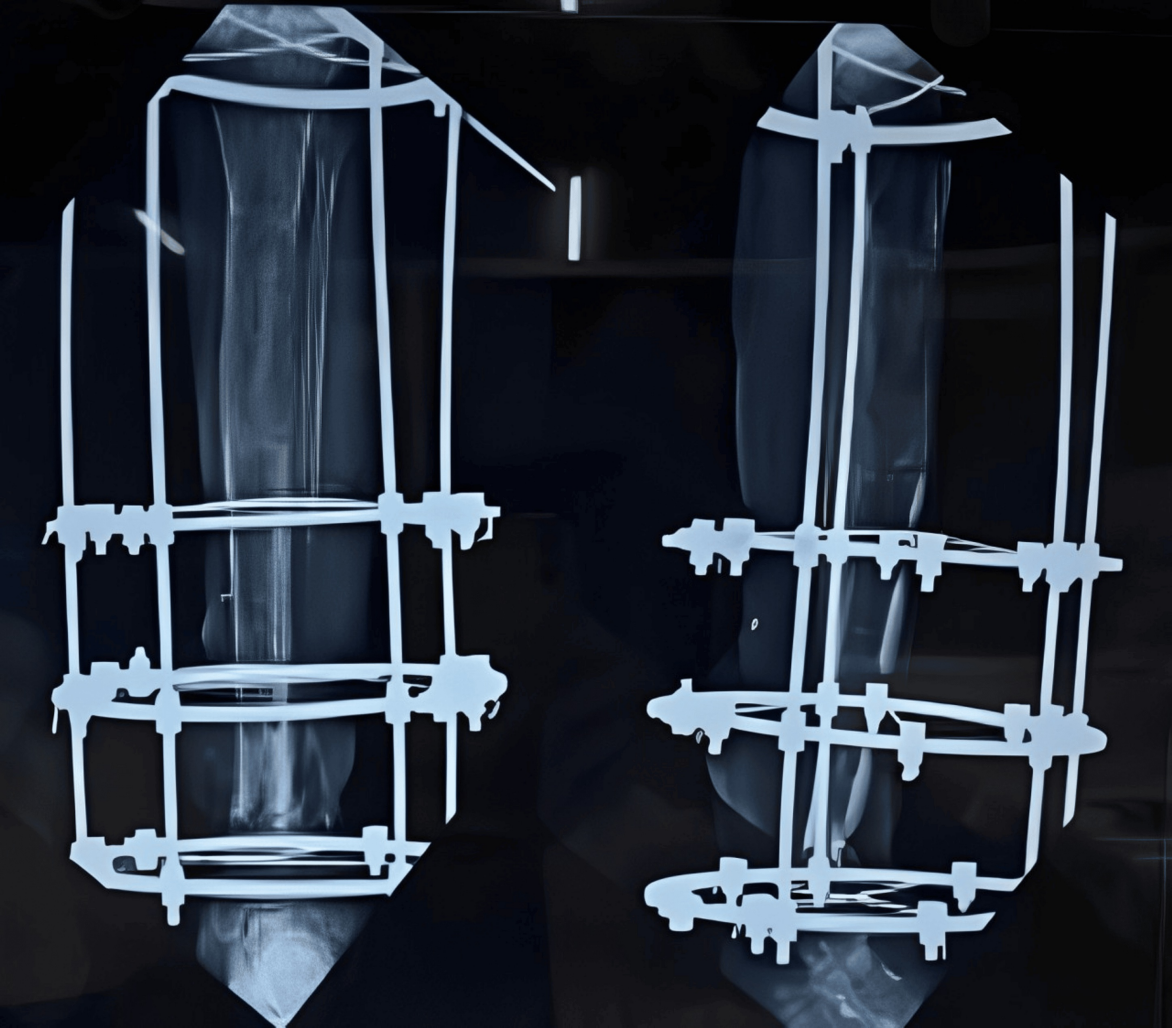
X-ray showing the consolidation and healing of the distraction site (after three months of stopping the distraction)

**Figure 11:**
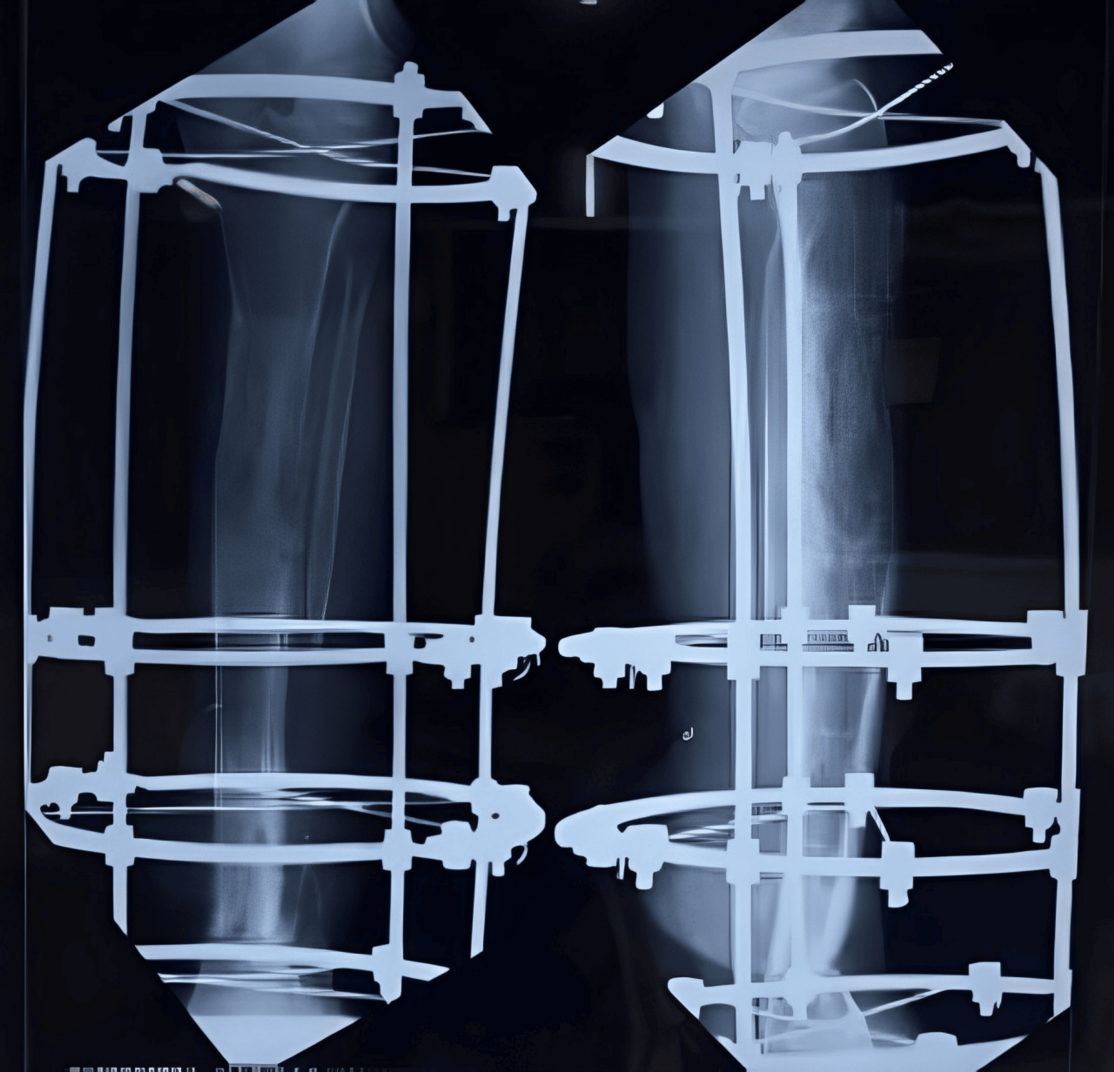
X-ray after six months of stopping the distraction showing a well-healing consolidation

Once the lengthening was completed after six months of gradual distraction, final X-rays were taken to confirm the completed lengthening (Figure 9). All distractions were then stopped, and the patient was encouraged to engage in full weight-bearing walking for increased duration to promote better healing at the distraction site and union at the distal nonunion site (Figure 10 and Figure 11).

**Figure 12:**
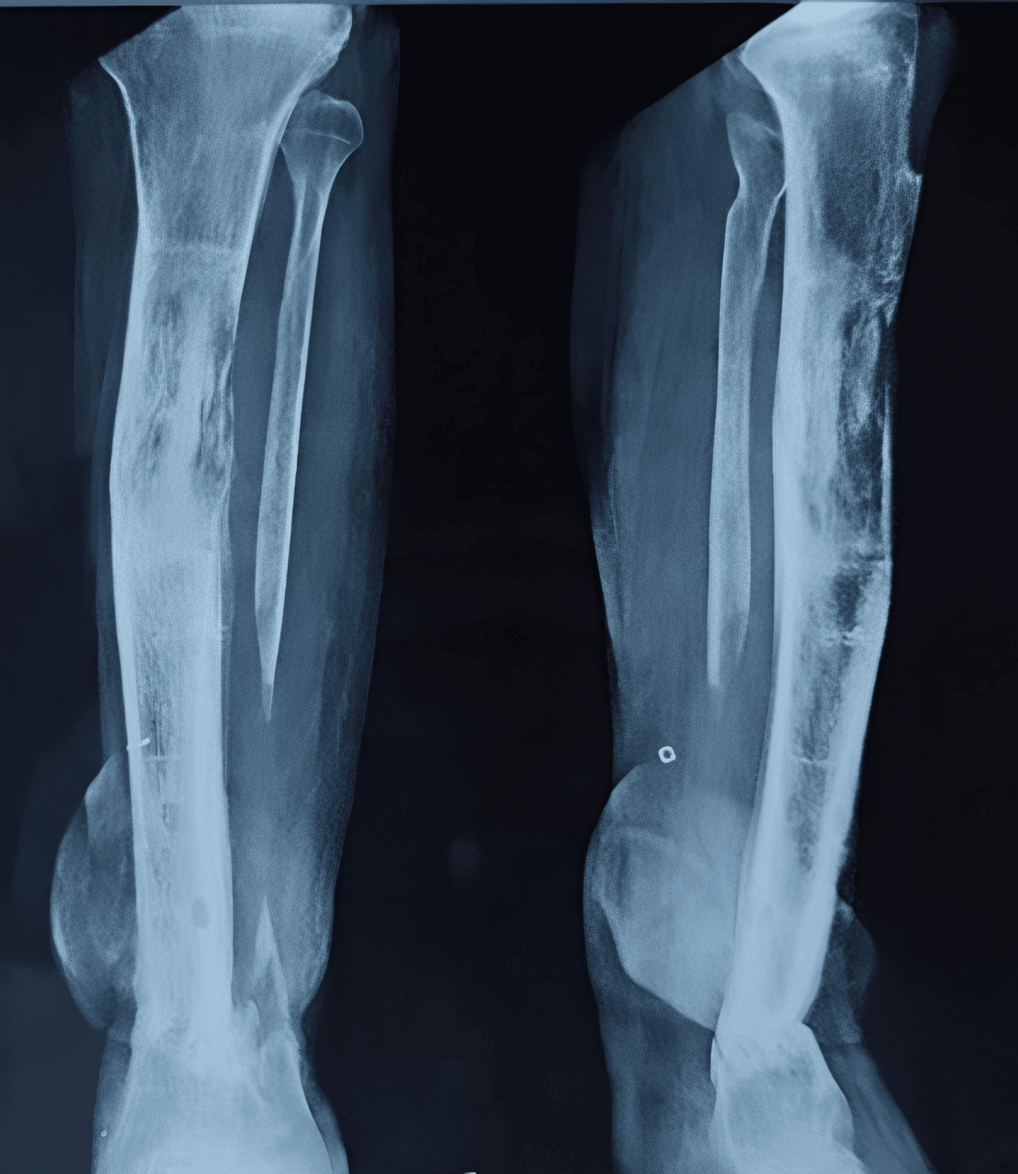
X-ray of the knee with the leg showing the complete healing at the nonunion site and consolidation at the distraction site

**Figure 13:**
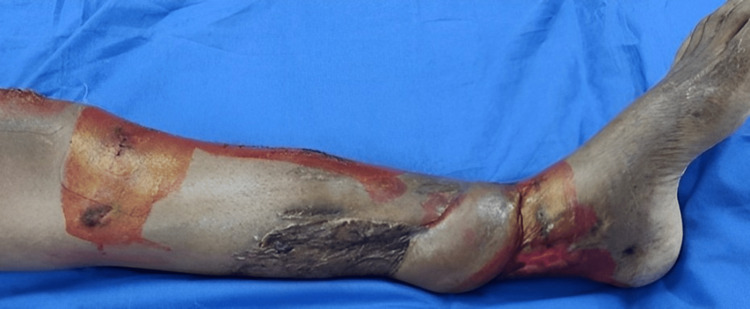
Clinical picture of the lower limb post-Ilizarov frame removal

Once healing was achieved at both the proximal and distal ends, the patient was tested under image intensification, and the Ilizarov fixator was removed under sedation and analgesia (Figure 12). There were no clinical signs of infection or limb length discrepancy (Figure 13).

**Figure 14:**
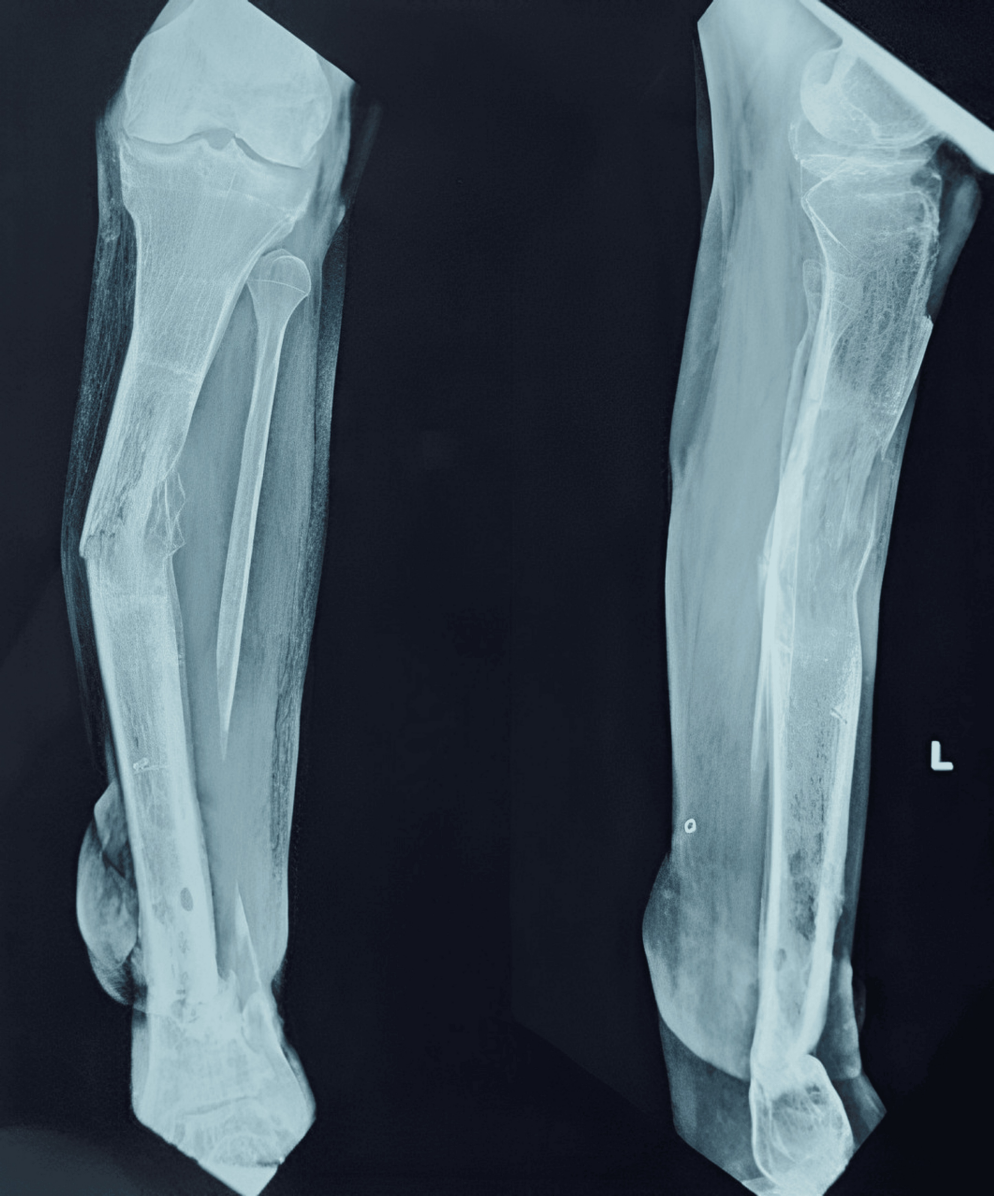
X-ray showing the fracture at the distal end of the distraction site

The patient was advised to bear weight with a custom-made thermoplastic brace for a period of 8-12 weeks. After two months, the patient presented to the outpatient department with pain and difficulty bearing weight on the previously operated leg. The patient reported no history of falls or trauma. A radiograph revealed a fracture at the distraction site (see Figure 14).

**Figure 15:**
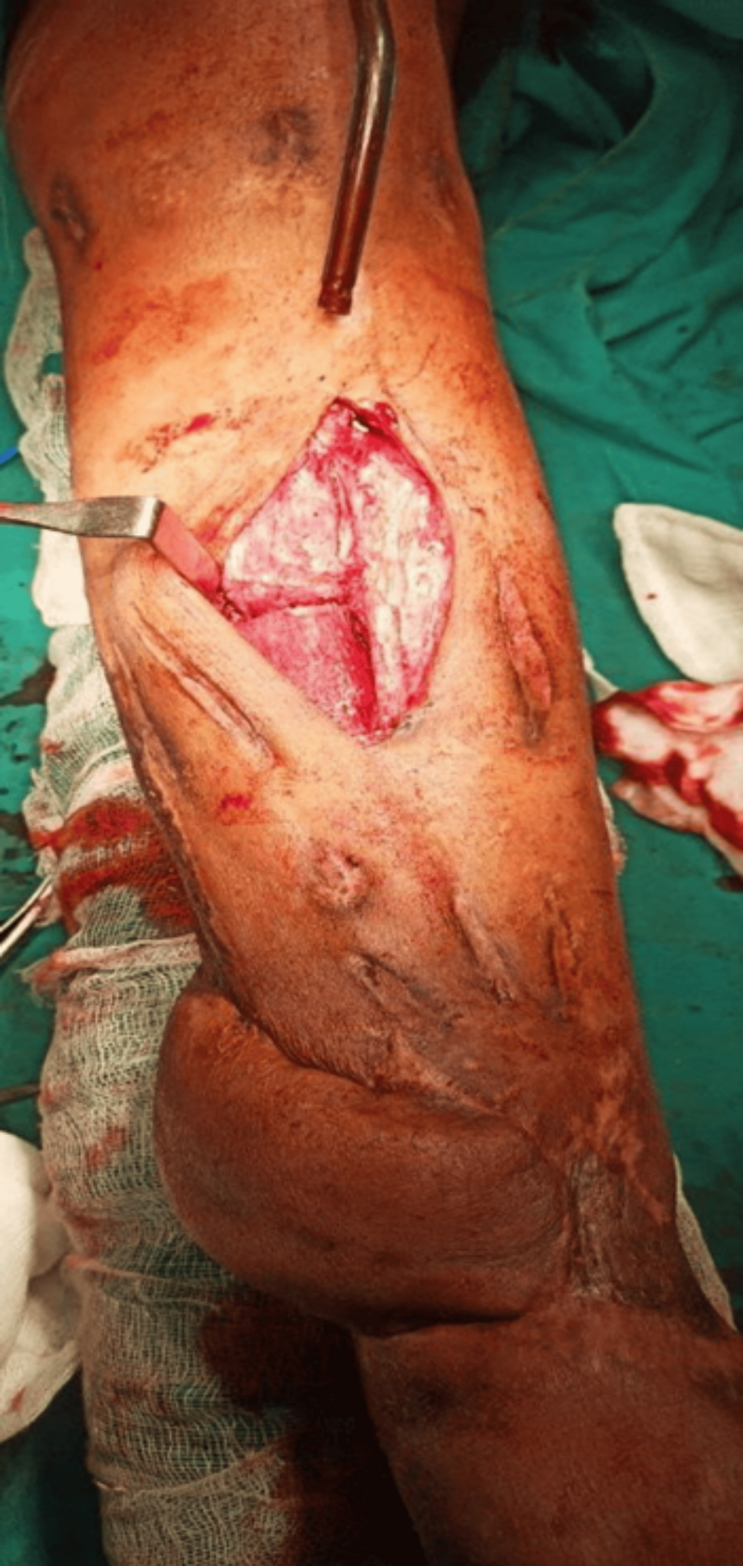
Clinical image showing the fracture at the distraction site (fracture was exposed and bone grafting was done)

**Figure 16:**
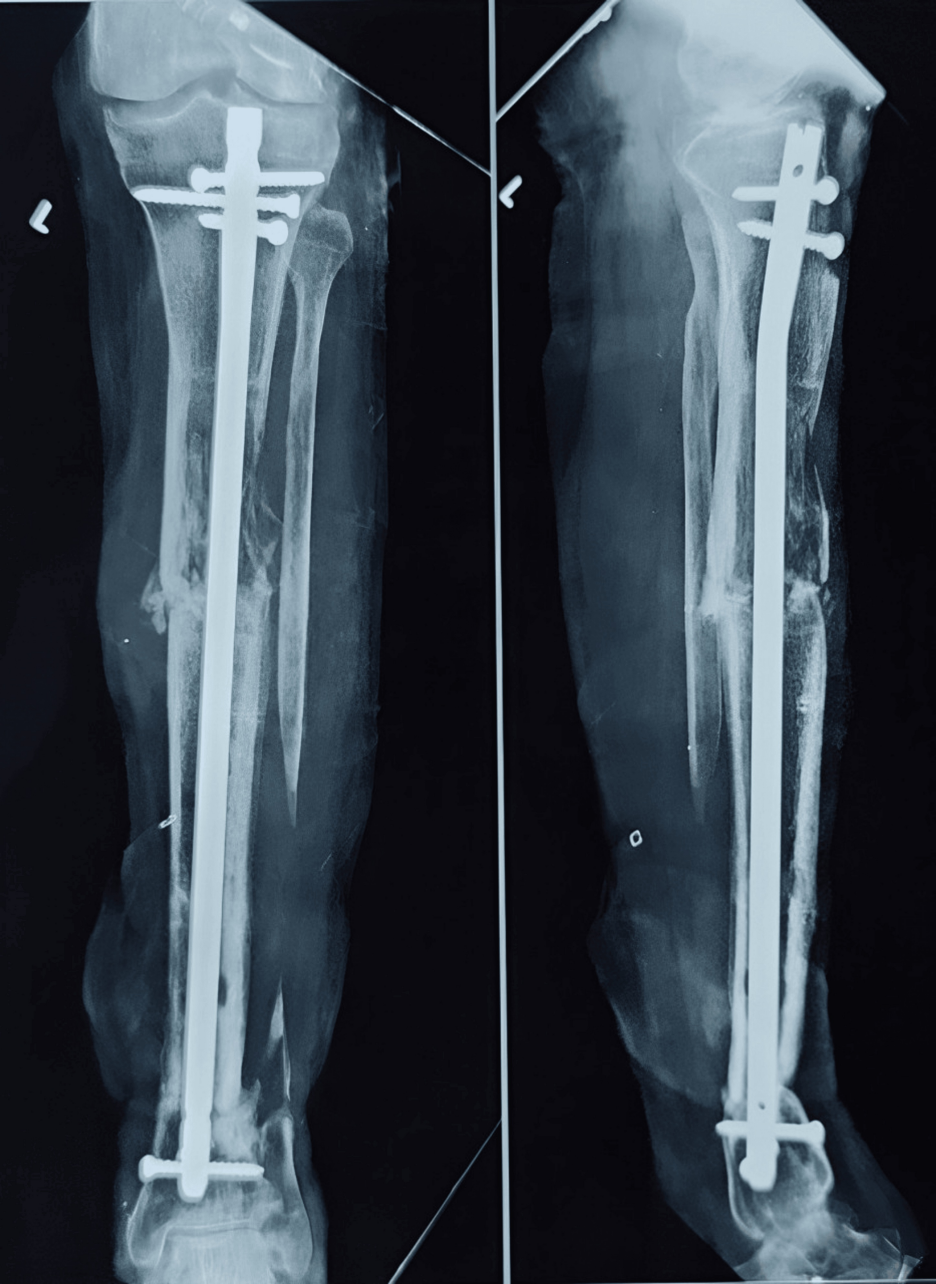
Postoperative X-ray showing the interlocking nail

The patient was advised to undergo surgical intervention involving a more stable fixation with an interlocking nail, along with autologous bone grafting. The prognosis was discussed with the patient, including the possibility of multiple surgeries (see Figure 15 and Figure 16).

**Figure 17:**
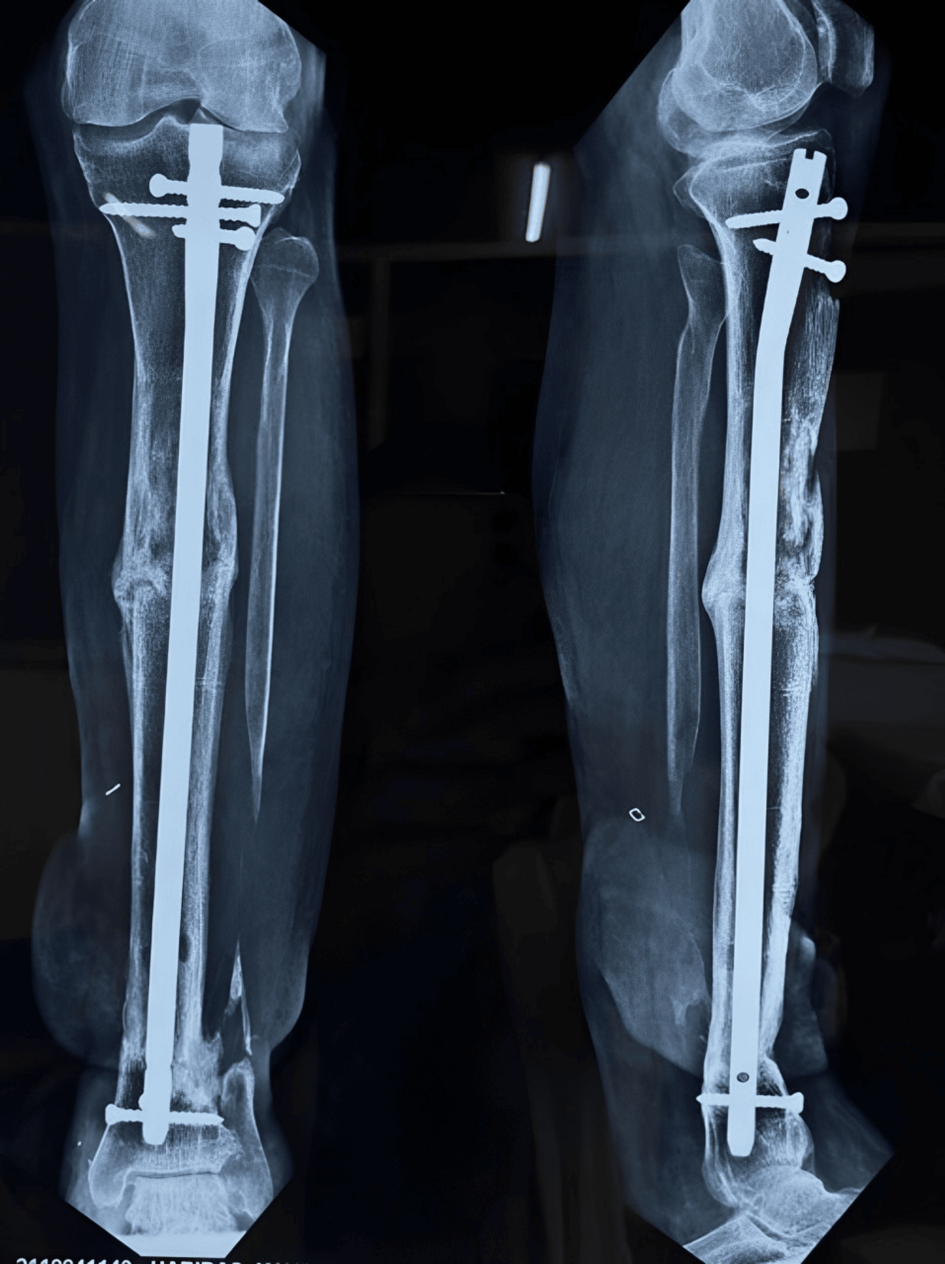
X-ray done at three months showing the initial signs of union

**Figure 18:**
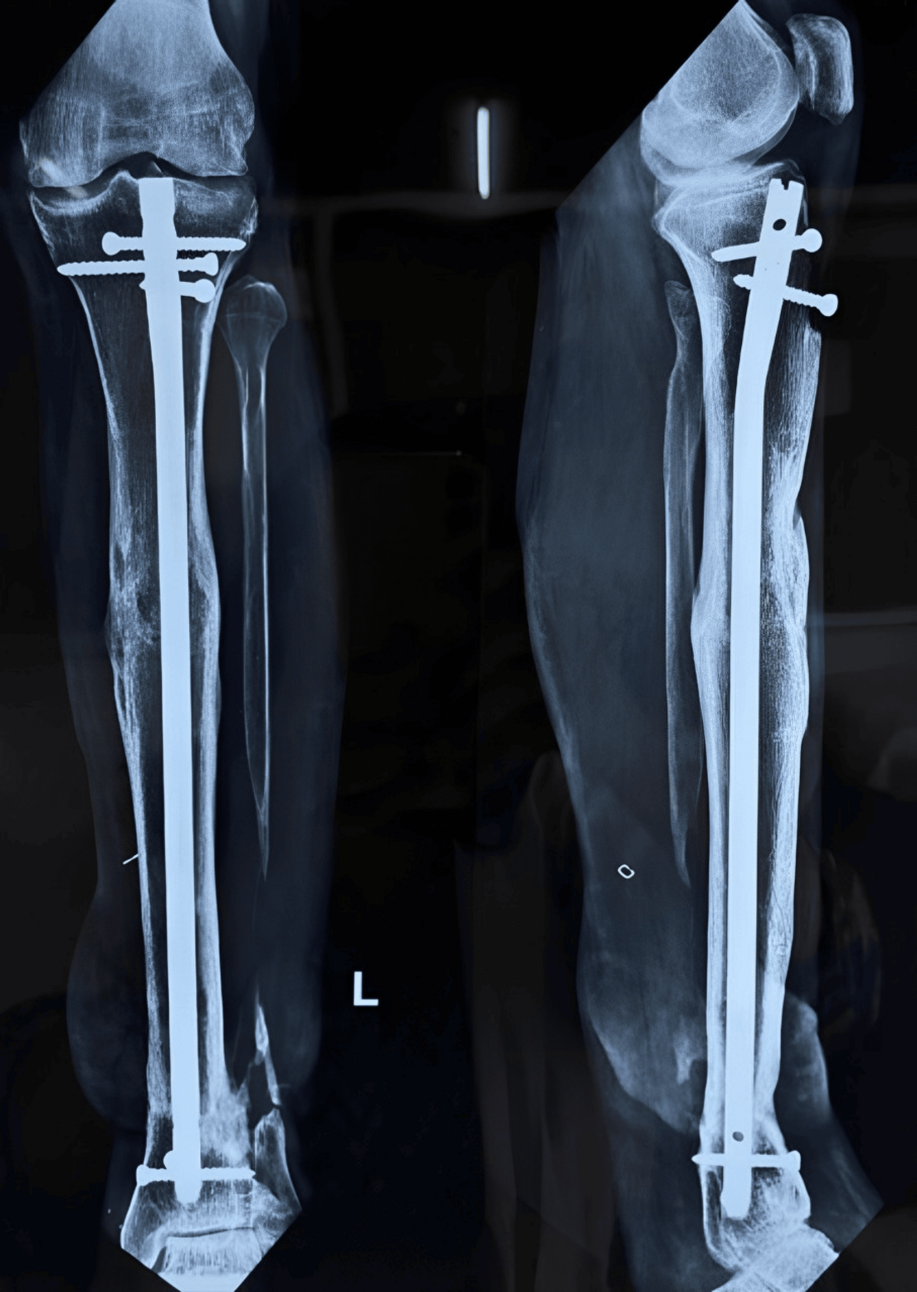
X-ray done at 12 months showing the complete union at the fracture site

**Figure 19:**
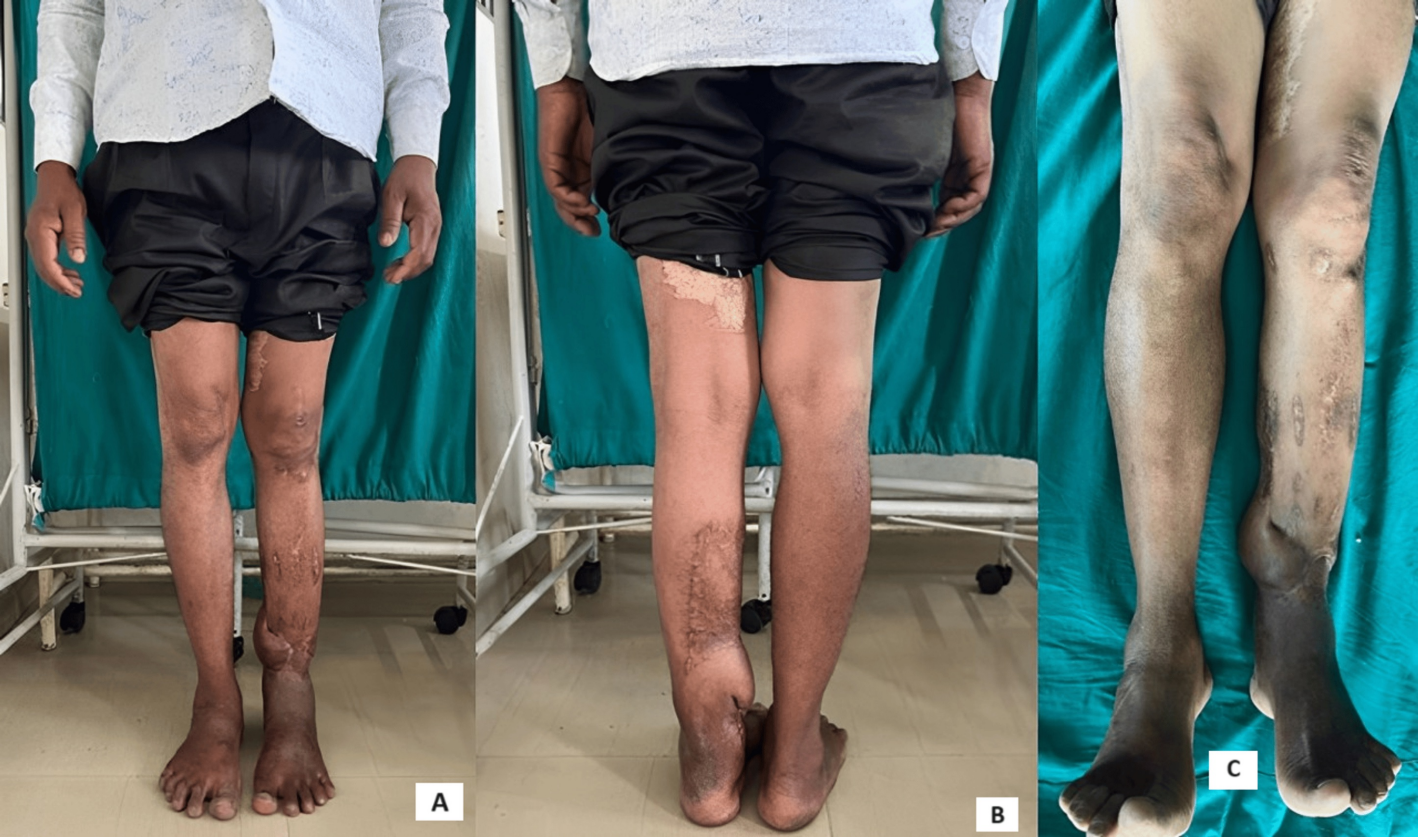
Clinical images of bilateral lower limbs showing no limb length discrepancy and no signs of infection at 12 months of follow-up A: Front view of bilateral lower limbs B: Back view of bilateral lower limbs C: Superior view of bilateral lower limbs

The patient was advised to partially bear weight while walking with walker support and to continue performing knee and ankle range-of-motion exercises. The patient was regularly monitored, and X-rays were conducted to assess the healing of the fracture (Figure 17 and Figure 18). There was no limb length discrepancy, and there were no clinical or hematological signs of infection (Figure 19).

## Discussion

Compound injuries resulting from high-energy trauma must be treated promptly to achieve optimal outcomes [8]. Delayed treatment can lead to complications such as nonunions and bone gaps. The management of infected comminuted distal tibia nonunion following high-energy trauma, such as from a road traffic accident, poses a significant challenge for orthopedic surgeons [9]. These cases necessitate a multidisciplinary approach and meticulous surgical planning. Successful management typically involves multiple staged procedures, including debridement, external or internal fixation, and soft tissue reconstruction [7]. Each stage of treatment is crucial for addressing various aspects of the injury and enhancing the likelihood of a successful outcome. External fixators, such as the Ilizarov fixator, are vital in managing resistant nonunions [10]. Despite thorough planning and management, challenges and complications may still arise, such as refractures at the nonunion site or the distraction site, though these are rare and have been previously documented [11]. A multicentric approach that includes orthopedic surgeons, plastic surgeons, and physiotherapists is essential for optimizing treatment and improving long-term outcomes. Additionally, patient commitment and dedication are critical for the success of long-duration treatments, including regular follow-ups to monitor progress and adjust management as needed.

## Conclusions

The successful management of challenging, resistant, and infected nonunions following high-energy trauma demands a comprehensive and integrated approach. This approach should encompass surgical expertise, wound care, infection management, and rehabilitation. By addressing each aspect of the injury sequentially and adapting the treatment plan as needed, favorable outcomes can be achieved. This approach not only restores function but also improves the patient's quality of life.
